# Ginsenoside Rh2 inhibits breast cancer cell growth viaERβ-TNFα pathway: Ginsenoside Rh2 inhibits breast cancer cell growth via ERβ-TNFα

**DOI:** 10.3724/abbs.2022039

**Published:** 2022-05-07

**Authors:** Kunjian Peng, Tiao Luo, Jijia Li, Jingjia Huang, Zizeng Dong, Jia Liu, Chaoqiong Pi, Zizeng Zou, Qin Gu, Ousheng Liu, Jian-Ting Zhang, Zhi-Yong Luo

**Affiliations:** 1 Department of Biochemistry and Molecular Biology Hunan Province Key Laboratory of Basic and Applied Hematology & Hunan Key Laboratory of Animal Models for Human Diseases School of Life Sciences Central South University Changsha 410008 China; 2 Department of Cell and Cancer Biology University of Toledo College of Medicine and Life Sciences Toledo OH 43614 USA; 3 Hunan Key Laboratory of Oral Health Research & Xiangya Stomatological Hospital & Xiangya School of Stomatology Central South University Changsha 410008 China; 4 Center of Stomatology Xiangya Hospital Central South University Changsha 410008 China.

**Keywords:** ginsenoside Rh2, estrogen receptor, TNFα, breast cancer, apoptosis

## Abstract

Ginsenoside Rh2 is one of rare panaxidiols extracted from *Panax ginseng* and a potential estrogen receptor ligand that exhibits moderate estrogenic activity. However, the effect of Rh2 on growth inhibition and its underlying molecular mechanism in human breast cells are not fully understood. In this study, we tested cell viability by MTT and colony formation assays. Cell growth and cell cycle were determined to investigate the effect of ginsenoside Rh2 by flow cytometry. The expressions of estrogen receptors (ERs), TNFα, and apoptosis-related proteins were detected by qPCR and western blot analysis. The mechanisms of ERα and ERβ action were determined using transfection and inhibitors. Antitumor effect of ginsenoside Rh2 against MCF-7 cells was investigated in xenograft mice. Our results showed that ginsenoside Rh2 induced apoptosis and G1/S phase arrest in MCF-7 cells. Treatment of cells with ginsenoside Rh2 down-regulated protein levels of ERα, and up-regulated mRNA and protein levels of ERβ and TNFα. We also found that ginsenoside Rh2-induced TNFα over-expression is through up-regulation of ERβ initiated by ginsenoside Rh2. Furthermore, ginsenoside Rh2 induced MCF-7 cell apoptosis via estrogen receptor β-TNFα pathway *in vivo*. These results demonstrate that ginsenoside Rh2 promotes TNFα-induced apoptosis and G1/S phase arrest via regulation of ERβ.

## Introduction

Natural products are potentially valuable sources for the development of new anti-cancer drugs [ 1, 2]. *Panax ginseng* Meyer, a traditional Chinese medicine, has been widely used for thousands of years in East Asia [3]. Ginsenosides are one of the major pharmacologically bioactive constituents extracted from *Panax ginseng* [4]. More than 100 ginsenoside compounds consisting of triterpene aglycones have been identified, which can be divided into three major categories: panaxidiols, panaxatriols, and oleanolic acid derivatives. [5]. Ginsenoside Rh2 is one of the rare panaxidiols and has been tested as novel agents to induce apoptosis or cell cycle arrest in a variety of cancer cells [ 6- 9]. Recently, it was reported that Rh2 treatment induces apoptosis in human promyelocytic leukemia HL-60 cells via up-regulation of tumor necrosis factor-alpha (TNFα) [10]. However, the precise molecular mechanism of the anti-tumor action of ginsenoside Rh2 remains unclear.

TNFα is a pleiotropic cytokine that plays a critical role in diverse cellular events, including cell proliferation, differentiation and apoptosis [11]. TNFα exists in both soluble and membrane bound forms. The soluble plasma form is a 17-kDa protein that forms a homotrimer for receptor activation and is cleaved from the membrane-bound form [12]. Tumor necrosis factor receptor 1 (TNFR1), as an important TNF alpha receptor, canonically stimulates a pro-death pathway through activating caspase-8 [13]. The anti-tumor activity of TNFα is now well established, and approximately 28% of cancers are susceptible to direct cell killing by soluble TNFα [12].

In humans there are two estrogen receptors (ERs), ERα and ERβ. Both are members of the nuclear receptor superfamily of hormone-inducible transcription factors that are involved in regulating many complex physiological processes [14]. Estrogen receptors include two domains: a ligand binding domain (LBD) that can interact with its hormonal activator such as 17β-estradiol, and a DNA binding domain (DBD) that can target DNA motif known as the estrogen responsive element (ERE) [15]. It is also noteworthy that ERα and ERβ form both homodimers and heterodimers in response to ligand binding, and both homo and heterodimers are capable of binding with EREs [ 16, 17]. Though the receptors have similar ligand-binding and DNA-binding domains, ERα and ERβ have some unique properties in terms of ligand selectivity and gene-targeted regulation [14]. In general, ERα is an oncogene that regulates genes involved in proliferation and metabolism, and its expression increases at the early stages of cancer [18]. On the contrary, ERβ, as a tumor suppressor gene, is thought to oppose the proliferative action of ERα in mammary cells, and ERβ levels are reduced during carcinogenesis and cancer progression [19]. Thus, ERα is a potential target for cancer therapy.

Though the precise molecular mechanism is unknown, the ginsenoside Rh2 has potential therapeutic effects against various cancers [ 6– 10]. It has been reported that ginsenoside Rh2 exhibits moderate but significant estrogenic activity that is 30% of the activity of 17β-estradiol [20].

In this study, we investigated the roles of estrogen receptors in ginsenoside Rh2 inhibition of tumor cell proliferation. Our results showed that ginsenoside Rh2 exerts significant anticancer activities by inducing apoptosis and cell cycle inhibition in breast cancer cells. Furthermore, we also indicated that ginsenoside Rh2 induces up-regulation of ERβ and down-regulation of ERα. Up-regulation of ERβ significantly increases the expression of TNFα that induces apoptosis of breast cancer cells.

## Materials and Methods

### Reagents and antibodies

20(S)-ginsenoside Rh2 (purity>98%) was purchased from the College of Chemistry, Jilin University (Changchun, China). It was dissolved in dimethyl sulphoxide (DMSO) at 50 mM and stored at −20°C. ICI182780, PHTPP Bafilomycin A1 were purchased from Santa Cruz Biotechnology (Santa Cruz, USA). MG132 was purchased from MedChem Express (Monmouth Junction, USA). DMSO was purchased from Sigma Aldrich (St Louis, USA).

Anti-ERβ (ab3576) antibody was purchased from Abcam (Cambridge, USA). Anti-TNFα (D5G9), anti-Bcl-XL (54H6), anti-Bax (D2E11), anti-Bad (D24A9), anti-Survivin (71G4B7), anti-Cleaved caspase-8 (11G10), anti-Cleaved PARP (D64E10), anti-cyclin D1 (92G2), anti-phospho-p38 (28B10), anti-p38 (D13E1) and anti-GAPDH (14C10) antibodies were purchased from Cell Signaling Technology (Danvers, USA). Anti-ERα (sc-8002) and anti-β-actin (sc-47778) antibodies were purchased from Santa Cruz Biotechnology.

### Cell culture and treatment

Human breast cancer MCF-7 and MDA-MB-231 cells were obtained from the Department of Pharmacology and Toxicology and IU Simon Cancer Center, Indiana University School of Medicine (Indianapolis, USA) and validated by short tandem repeat (STR) profiling. Cells were cultured in our laboratory for less than 6 months in DMEM (Life Technologies, Gaithersburg, USA) and DMEM/F12 (Life Technologies), respectively. Maintenance media were supplemented with 10% FBS (Hyclone, South Logan, USA). Cells were maintained in humidified atmosphere with 5% CO _2_ at 37°C.

### MTT assay

Cells were plated in 96-well plates at a density of 3000 cells per well in DMEM supplemented with 10% FBS. Twelve hours prior to treatment, media were replaced by DMEM supplemented with different concentrations (0, 10, 20, 30, 40, 50, 60, 70 and 80 μM) of ginsenoside Rh2, and cell viability was determined after 24, 48 and 72 h of culture. The cells were further incubated with MTT solution (5 mg/mL; Sigma Aldrich, St Louis, USA) for an additional 4 h at 37°C. After the media were replaced by DMSO, the absorbance of each well was measured at 570 nm with a microplate reader.

### CCK8 assay

Cells were plated in 96-well plates at a density of 1000 cells per well in DMEM supplemented with 10% FBS. Cell viability was determined after 24, 48, 72 and 96 h of culture. The cells were further incubated with CCK8 reagent (MedChem Express) for an additional 2 h at 37°C. The absorbance of each well was measured at 450 nm with a microplate reader.

### Colony formation assay

MCF-7 and MDA-MB-231 cells were seeded into 6-well plates at 800 cells per well and cultured for 14 days with different concentrations (0, 20, 30 and 40 μM) of ginsenoside Rh2. Cells were fixed with 4% paraformaldehyde for 20 min and stained with 0.1% crystal violet for 30 min. The images of colonies in the wells were captured and the number of colonies in each well was counted using Image J software. Experiments was performed in triplicate and repeated three times.

### Cell apoptosis assays

The cell apoptosis assays included Annexin V/propidium iodide (PI) fluorescent-activated cell sorting (FACS) assay and histone-bound DNA apoptosis enzyme-linked immunosorbent assay (ELISA) assay.

In FACS assay, cells were seeded into 6-well plates at 2.5×10 ^5^ cells/well. MCF-7 cells were treated with different concentrations (0, 30, 40, 45 and 50 μM) of ginsenoside Rh2, and then stained using the Annexin V-FITC/PI Apoptosis Detection Kit (Beyotime Biotechnology, Shanghai, China) according to the instructions provided with the kit. Briefly, 5 μL of Annexin V-FITC and 5 μL of PI solution were added to the cell suspension, gently mixed, and incubated for 15 min at room temperature. Then, 400 μL of binding buffer was added to get the samples, and the samples were analyzed on a BD FACScan flow cytometer (BD Biosciences, San Jose, USA).

In histone-bound DNA apoptosis enzyme-linked immunosorbent assay, MCF-7 cells were plated into 96-well plates at 4000 cells per well and treated with different concentrations (0, 40, 45 and 50 μM) of ginsenoside Rh2 for 48 h. The apoptosis of MCF-7 cells were measured using apoptosis detection kits (Boehringer Mannheim, Mannheim, Germany) according to the manufacturer′s instructions.

### Cell cycle analysis

MCF-7 cells were seeded into 6-well plates at 2.5×10 ^5^ cells/well and were treated with different concentrations (0, 30, 40, 45 and 50 μM) of ginsenoside Rh2. The cells were collected by centrifugation at 100×g for 5 min, fixed in 70% ice-cold ethanol at 4°C for 12 h. Fixed cells were washed twice with PBS and resuspended in 1 mL of PBS containing 2.5 μg/mL ribonuclease and 50 μg/mL propidium iodide. After incubation in the dark for 30 min at room temperature, cells were analyzed using a BD FACScan flow cytometer (BD Biosciences). A total of 10,000 events were acquired for analysis using Flowjo software.

### Western blot analysis

Whole-cell extracts were prepared using RIPA buffer containing protease and phosphatase inhibitor cocktails (87785, 78420; Thermo Scientific, Waltham, USA), and the protein concentrations were determined by using bicinchoninic acid (BCA) assay reagent (Thermo Scientific). Equal amounts of protein samples (30 μg) were separated by SDS-PAGE and transferred to polyvinylidene difluoride (PVDF) membranes. Membranes were blocked in PBS with 0.1% Tween-20 (1×PBST) containing 5% fat-free milk at room temperature for 2 h and then incubated with primary antibodies overnight at 4°C. Then, membranes were washed with 1×PBST for 3 times followed by 2 h of incubation with the corresponding HRP-conjugated secondary antibodies at room temperature. Membranes were washed with 1×PBST for 3 times and visualized using enhanced chemiluminescence kit (Invitrogen, Carlsbad, USA). Protein bands were analyzed using the Gel Doc XR ChemiDoc imaging system (Bio-Rad, Hercules, USA) and quantified using Quantity One software (Bio-Rad).

### qRT-PCR analysis

Total RNA was extracted from MCF-7 cells using Trizol reagent (Life Technologies Corporation, Carlsbad, USA) and reverse transcripted using the mRNA Reverse Transcription Kit (Thermo Scientific) according to the manufacturer’s instructions. The mRNA expression levels of ERα, ERβ and TNFα were measured using SYBR Green RNA Kit (Applied Biosystems, Foster City, USA) according to the manufacturer’s instructions. The PCR cycle conditions were as follows: 95°C for 1 min and 40 cycles at 95°C for 15 s, 56°C for 20 s, and 72°C for 20 s. The primers used were as follows: *TNFα* forward: 5′-TGAGCACTGAAAGCATGATC-3′ and reverse: 5′-TCACAGGGCAATGACCCAAA-3′; *ERβ* forward: 5′-AGCACGGCTCCATATACATACC-3′ and reverse: 5′-TGGACCACTAAAGGAGAAAGGT-3′; *ERα* forward: 5′-CCCACTCAACAGCGTGTCTC-3′ and reverse: 5′-CGTCGATTATCTGAATTTGGCCT-3′; *GAPDH* forward: 5′-GTCGGAGTCAACGGATTTGG-3′ and reverse: 5′-ACACCCATGACGAACATGGG-3′.

### Cell transfection

Small interfering RNA (siRNAs) and the negative control were synthesized by GenePharma (Shanghai, China). Cells were plated into 6-well plates at 2.5×10 ^5^ cells/well and cultured for 24 hours, and then transfected with 50 nM siRNA or 2 μg plasmid using the Lipofectamine 2000 transfection reagent (Invitrogen, Carlsbad, USA) according to the manufacturer′s protocol. Transfected cells were cultured for appropriate time before the subsequent assays. The sequences of siRNAs were as follows: Control-siRNA, 5′-GCGCGCUUUGUAGGAUUCGdTdT-3′; ERα-siRNA, 5′-GACUUGAAUUAAUAAGUGAdTdT-3′; ERβ-siRNA-1, 5′-GCAUGGAACAUCUGCUCAAdTdT-3′; ERβ-siRNA-2, 5′-GCUGCUGGAGAUGCUGAAUdTdT-3′.

### Luciferase assay

The TNF promoter was amplified using primers 5′-ACTCGAGGCCGCCAGACTGCTGCAGGGGA-3′ and 5′-ACCATGGAGAGGGTGGAGCCGTGGGTCA-3′ which contained tails for cloning into pGL4.10 (Promega, Madison, USA). Approximately 10 ^5^ MCF7 cells were grown to 95% confluency in 0.5 mL of DMEM in 24-well plates. Cells were transiently transfected using Lipofectamine 2000 transfection reagent (Invitrogen) according to the manufacturer’s protocol. Seventy-two hours after treatment with ginsenoside or DMSO, the luminescence was measured using the One-Lumi™ II Firefly Luciferase Reporter Gene Assay Kit (Beyotime Biotechnology) with a microplate luminometer (Berthold, Pforzheim, Germany). Luciferase activities are representative of at least three independent transfection experiments each at least in triplicate.

### Survival analysis

Survival analysis was performed according to a previous report [21]. Survival analysis of ERβ was performed for longer overall survival and disease-free survival by The Kaplan Meier plotter ( https://kmplot.com/analysis/), whose sources for the databases include GEO, EGA, and TCGA. It splits breast cancer patients by ‘Auto select best cutoff’ and generates Kaplan-Meier plots to visualize correlation between ERβ expression and survival.

### Nude mouse xenograft model

Female athymic BALB/c nude mice (6–8 weeks) were purchased from Central South University (Changsha, China) and maintained under specific pathogen-free conditions at the Department of Laboratory Animals, Central south University. The mice were randomly divided into 3 groups ( *n*=7) and 4×10 ^6^ MCF-7 cells were subcutaneously injected into the flank region of each mouse. In detail, mice in the negative control group were intraperitoneally injected with PEG400 and normal saline; mice in the ginsenoside Rh2 group were intraperitoneally injected with 20 mg/kg/day ginsenoside Rh2; mice in the positive control group were intraperitoneally injected with 3 mg/kg/day cisplatin. The subcutaneous tumor size was measured with a caliper and the tumor volume was calculated by the formula (length)×(width ^2^)/2. Mice were sacrificed at the 21st day after treatment. Tumors were harvested and processed for western blot analysis and immunohistochemical analysis. The research protocol was performed strictly in accordance with the Institutional Guidelines for the Care and Use of Laboratory Animals of the Central South University.

### TUNEL assay

Detection and quantification of apoptosis were performed using One-Stop Biotin-Based TUNEL Kit (KALANG, Shanghai, China). Histological sections of tumors were labeled with terminal deoxynucleotidyl transferase-mediated dUTP nick end labeling (TUNEL) according to the manufacturer′s instructions. TUNEL positive cells were counted using Image J software.

### Statistical analysis

All experiments were repeated three times. Data are presented as the mean±SD. Statistical analyses were performed by using Graphpad statistical software. The *P*-values were calculated using a one-way analysis of variance (ANOVA). *P*<0.05 was considered as statistically significant.

## Results

### Ginsenoside Rh2 inhibits viability of human breast cancer cells

**Figure 1:**
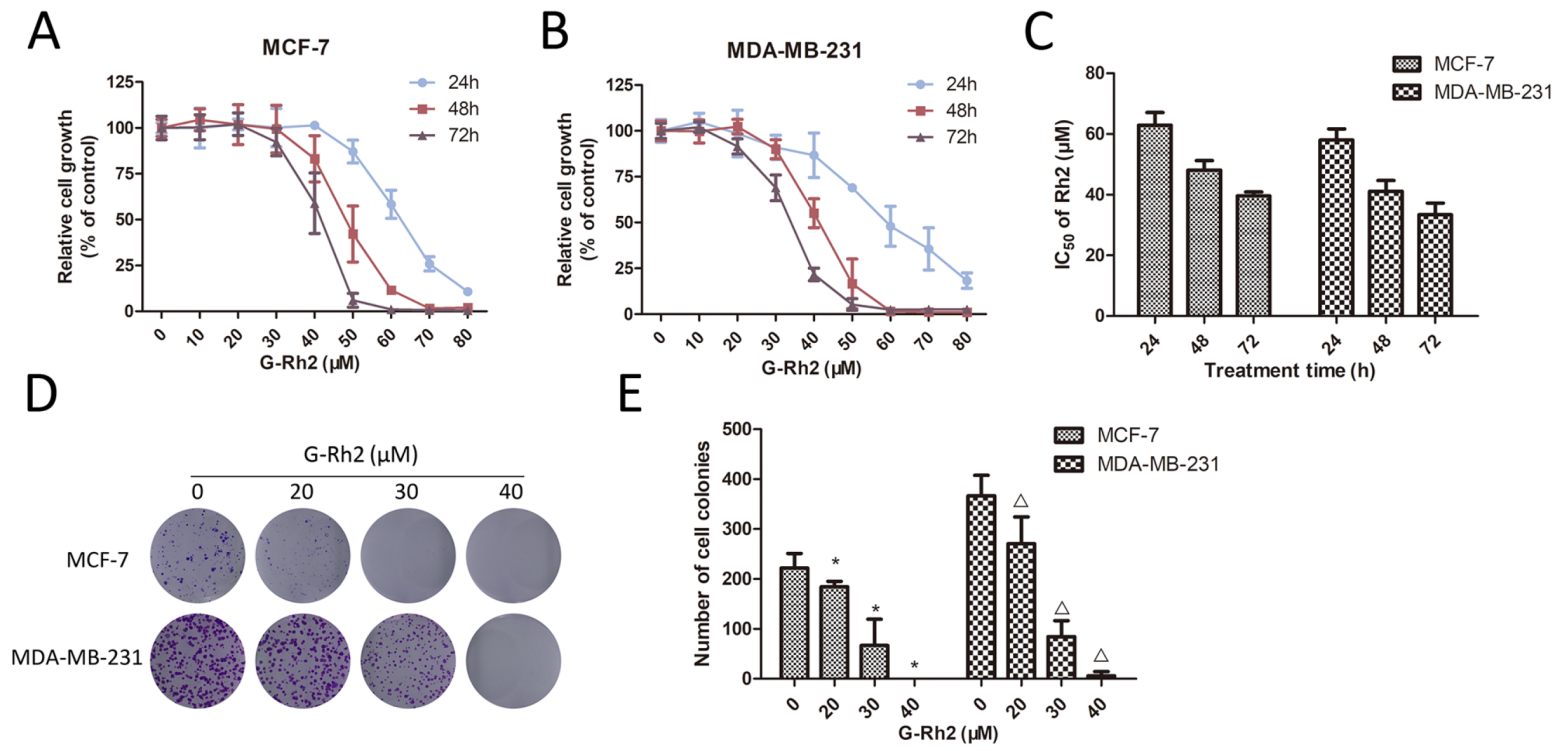
Figure1 Effects of ginsenoside Rh2 on proliferation of breast cancer cells (A) MCF-7 and (B) MDA-MB-231 cells were treated with different concentrations of Rh2 for 24, 48 and 72 h, and cell proliferation was detected by MTT assay. (C) The IC50 values of ginsenoside Rh2 in MCF-7 and MDA-MB-231 cell lines. (D) MCF-7 and MDA-MB-231 cells were treated with various concentrations of ginsenoside Rh2 for 14 days and the representative images of colony formation experiments were shown. (E) The quantitative analysis of colony formation assay. Data are presented as the mean±SD of three independent experiments. *P<0.05 versus control group.

The inhibitory effects of ginsenoside Rh2 on the viability and proliferation of human breast cancer MCF-7 and MDA-MB-231 cells were assessed by MTT assay. As shown in Figure 1A–C, ginsenoside Rh2 concentration-dependently inhibited the viability of both cells, with the IC _50_ values ranging from 40 to 63 μM for MCF-7 cells and from 33 to 58 μM for MDA-MB-231 cells with different treatment durations. Ginsenoside Rh2 also dose-dependently inhibited colony formation efficiency of MCF-7 and the MDA-MB-231 cells ( Figure 1D,E). These results suggest that ginsenoside Rh2 significantly inhibits viability and proliferation of MCF-7 and MDA-MB-231 cells.

### Ginsenoside Rh2 induces apoptosis and inhibits G1/S phase transition

**Figure 2:**
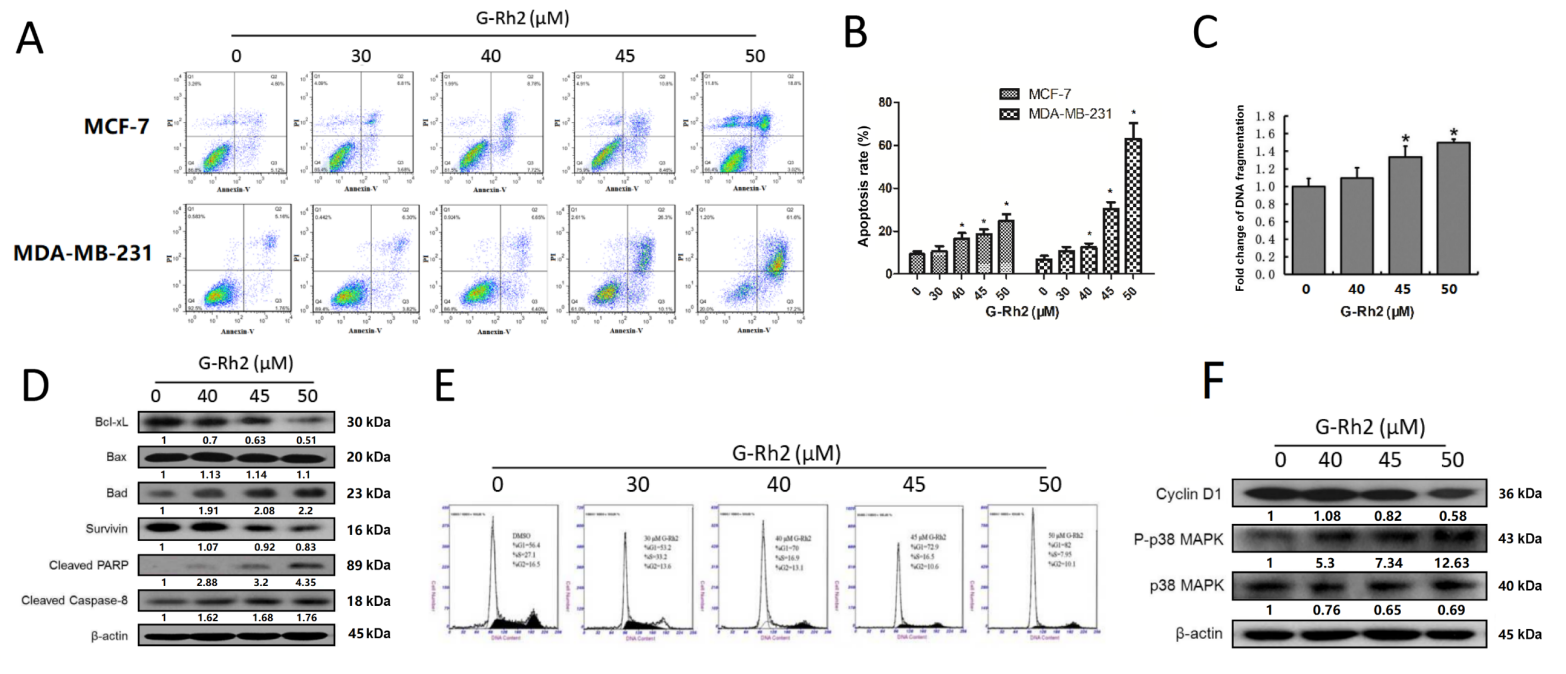
Figure2 Ginsenoside Rh2-induced apoptosis and G1/S phase arrest in breast cancer cells (A) Cell apoptosis of MCF-7 and MDA-MB-231 cells treated with different concentrations of Rh2 for 48 h was detected by flow cytometry after PI/Annexin V staining. (B) Quantification of apoptosis. Data are presented as the mean±SD of three independent experiments. *P<0.05 versus control group. (C) Apoptosis of MCF-7 cells treated with different concentrations of Rh2 for 48 h was detected by histone DNA ELISA. Fold change of DNA fragmentation was calculated. Data are presented as the mean±SD of three independent experiments. *P<0.05 versus control group. (D) MCF-7 cells were treated with different concentration of Rh2, and then protein samples were collected at 48 h for western blot analysis. (E) Cell cycle distribution of MCF-7 cells treated with different concentrations of Rh2 for 48 h was detected by flow cytometry after PI staining. (F) MCF-7 cells were treated with different concentrations of Rh2, and then protein samples were collected at 48 h for western blot analysis.

We next determined if ginsenoside Rh2 inhibits cell viability through the induction of apoptosis of breast cancer cells. Annexin V staining and FACS assay showed that ginsenoside Rh2 dose-dependently increased the number of Annexin V positive cells ( Figure 2A,B), indicating that it activated the apoptosis of both MCF-7 and MDA-MB-231 cells. The induction of apoptosis by ginsenoside Rh2 was also confirmed by histone DNA apoptosis enzyme-linked immunosorbent assay in MCF-7 cells ( Figure 2C).

To further investigate Rh2-induced apoptosis, the expressions of the key proteins in apoptosis process including Bcl-XL, Bax, Bad, survivin, cleaved caspase-8 and cleaved PARP were determined by western blot analysis following treatment of MCF-7 cells with different concentrations of ginsenoside Rh2. As shown in Figure 2D, ginsenoside Rh2 dose-dependently increased the expression of pro-apoptotic protein Bad and survivin reduced the expressions of anti-apoptotic proteins Bcl-XL and survivin. Both cleaved caspase-8 and PARP were increased by ginsenoside Rh2 treatment, confirming the induction of apoptosis by ginsenoside Rh2.

Next, cell-cycle analysis of MCF-7 cells was performed after ginsenoside Rh2 treatment . As shown in Figure 2E, ginsenoside Rh2 dose-dependently increased the proportion of cells in G1 phase. To further understand the role of ginsenoside Rh2 in G1/S phase transition, its effect on the expressions of proteins related to cell cycle progression was determined. As shown in Figure 2F, cyclin D1, a G1-specific cyclin which is associated with CDK4 or CDK6 and promotes restriction point progression during G1 phase [22], was repressed after treatment with ginsenoside Rh2. The expression and phosphorylation of p38, a tumor suppressor by negatively regulating cell cycle progression and inducing apoptosis [23], were also examined. As shown in Figure 2F, ginsenoside Rh2 dose-dependently increased the level of the phosphorylated p38. Thus, ginsenoside Rh2 may cause G1 phase arrest by activating p38 and reducing cyclin D1 expression.

### Ginsenoside Rh2 induces over-expressions of ERβ and TNFα but down-regulates ERα

**Figure 3:**
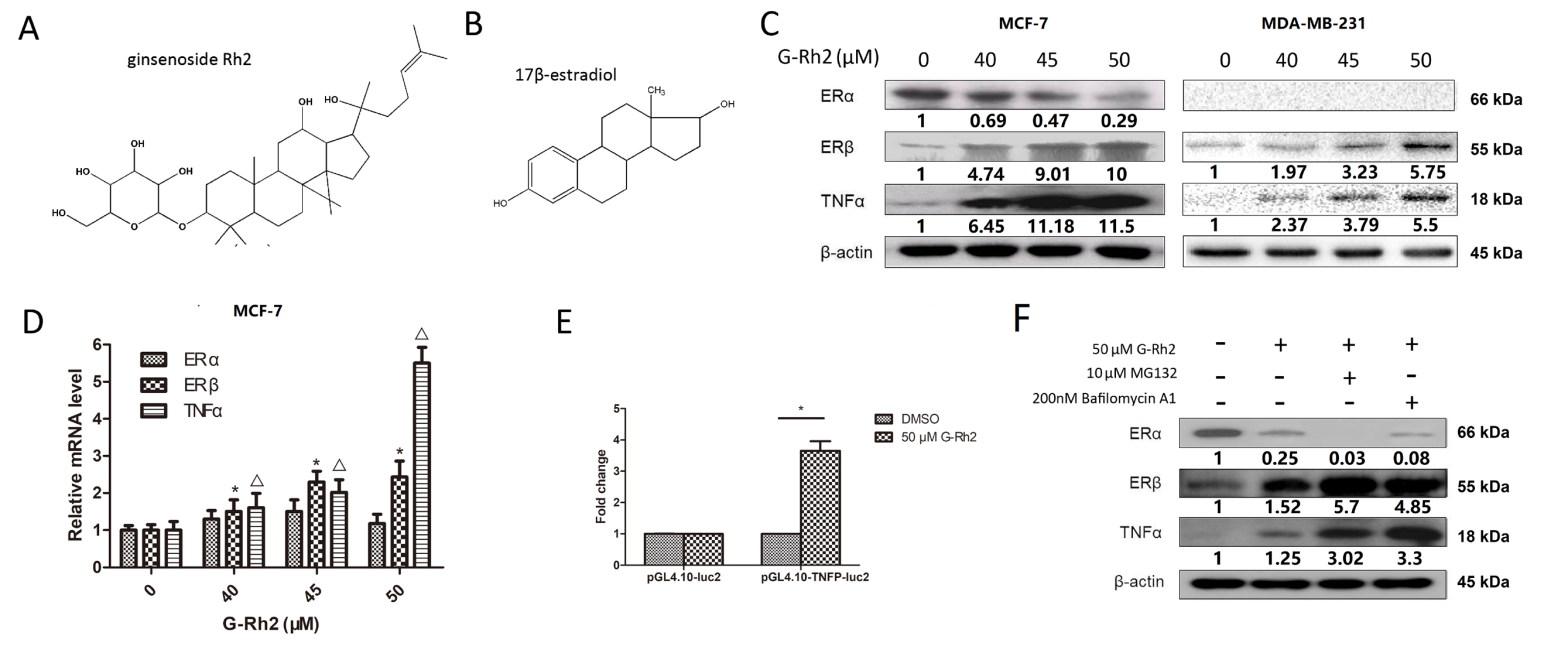
Figure3 Effects of ginsenoside Rh2 on the expressions of ERα, ERβ and TNFα in breast cancer cells Chemical structures of the ginsenoside Rh2 (A) and 17β-estradiol (B). Cells were incubated with 0, 40, 45, 50 μM ginsenoside Rh2 for 48 h and then harvested. The levels of proteins (C) and mRNAs (D) were determined by western blot and qRT-PCR analyses, respectively. Data are presented as the mean±SD of three independent experiments. *P and ΔP<0.05 versus control group. (E) The TNFα promoter luciferase reporter gene plasmid was transfected into MCF-7 cells in the presence or absence of 50 μM ginsenoside for 72 h. The activity of TNFα promoter was determined using luciferase reporter gene system. Data are presented as the mean±SD of three independent experiments. *P<0.05 versus control group. (F) Cells were treated with 10 μM MG132 or 200 nM Bafilomycin A1 in the presence or absence of 50 μM ginsenoside Rh2 for 48 h, protein samples were collected for western blot analysis.

It has been reported that TNFα plays an important role in Rh2-induced apoptosis [10]. As mentioned above, ginsenoside Rh2 also exhibits moderate estrogenic activity and has some similarities in chemical structure with 17β-estradiol ( Figure 3A,B). As a panaxidiol, ginsenoside Rh2 has a hydroxyl group at C-20 and the ring D with a hydroxyl group at 17β-position has similarity with 17β-estradiol [20]. Based on these previous observations, the effects of ginsenoside Rh2 on expressions of ERα, ERβ and TNFα in MCF-7 and MDA-MB-231 cells were tested by western blot analysis. As shown in Figure 3C, ginsenoside Rh2 dose-dependently down-regulated ERα protein expression, but up-regulated the expressions of ERβ and TNFα proteins. Next, we explored whether ginsenoside Rh2 treatment affects the mRNA levels of ERα, ERβ and TNFα by qRT-PCR. As shown in Figure 3D, ginsenoside Rh2 also dose-dependently increased the mRNA levels of ERβ and TNFα. However, Rh2 treatment had no effect on the ERα mRNA level. In addition, ginsenoside Rh2 enhanced the activity of TNFα promoter luciferase report gene ( Figure 3E), indicating that Rh2 directly induced the up-regulation of TNFα.

We next evaluated whether the Rh2 treatment affects the degradation of ERα, ERβ and TNFα proteins through the autophagy or ubiquitin-proteasome system by using autophagy inhitibitor Bafilomycin A1 and proteasome inhibitor MG-132. Figure 3F showed that MG-132 and Bafilomycin A1 increased the accumulation of ERβ and TNFα proteins in the cells, while MG-132 or Bafilomycin A1 down-regulated the protein level of ERα in Rh2-treated MCF-7 cells, suggesting that Rh2-induced down-regulation of ERα is independent of autophagy or the ubiquitin-proteasome system.

### Tumor suppressor gene *ERβ* regulates the over-expression of TNFα

**Figure 4:**
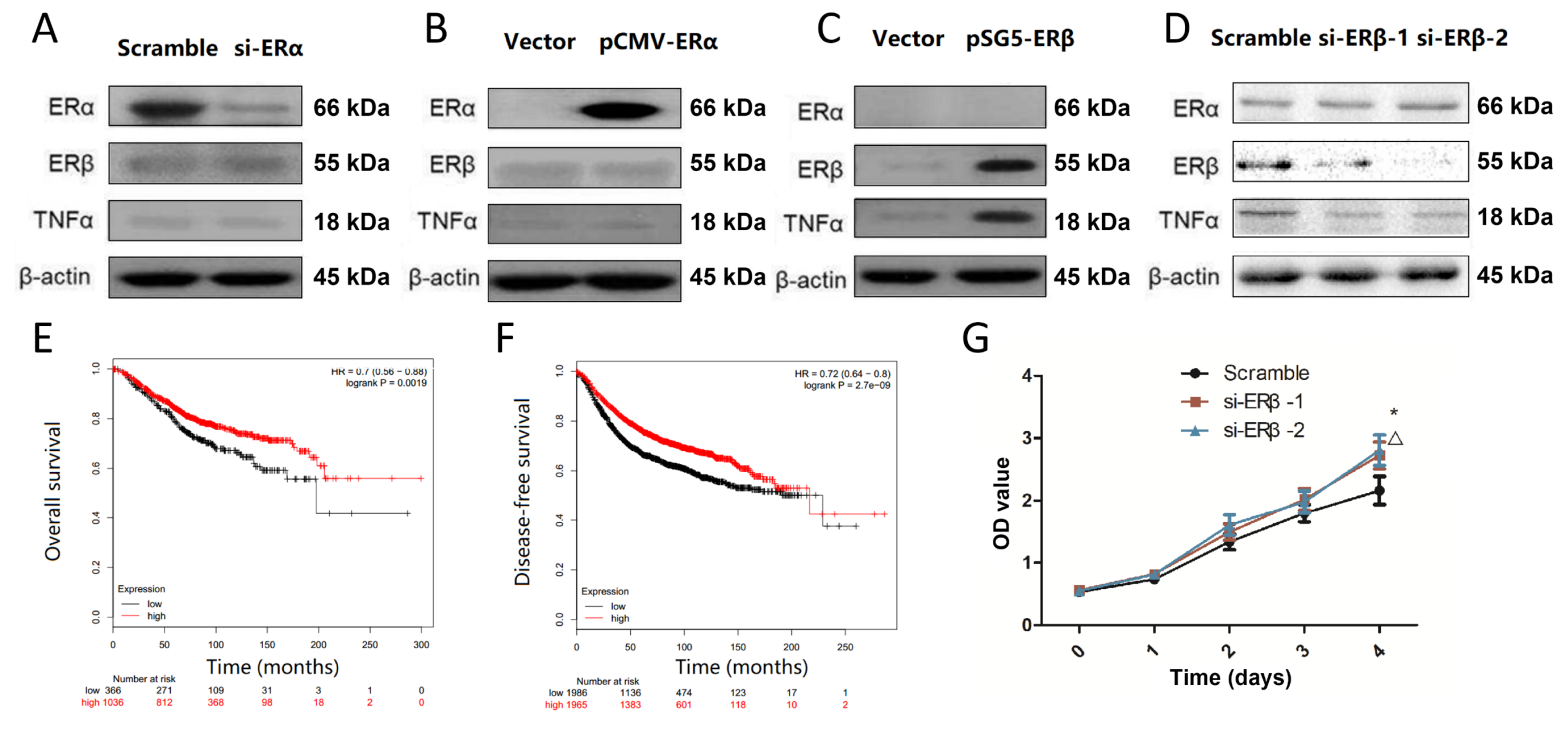
Figure4 ERβ plays an anti-cancer role in breast cancer MCF-7 cells were transfected with ERα siRNA (A) or ERβ siRNA (D). MDA-MB-231 cells were transfected with pCMV-ERα (B) or pSG5-ERβ (C) for 72 h followed by determination of protein levels of ERα, ERβ and TNFα by western blot analysis. Kaplan–Meier plots showing the association between ERβ expression and either overall survival (E) or disease-free survival (F) in ESCC patients. (G) MCF-7 cells were transfected with different ERβ si-RNAs, and then subject to CCK8 assay.

In order to determine whether Rh2-induced TNFα increase is a consequence of ER alteration, we evaluated TNFα protein in ER-positive MCF-7 cells after knockdown of *ERα* and *ERβ*, and in ER-negative MDA-MB-231 cells after over-expression of ERα and ERβ, respectively. As shown in Figure 4A,B, ERα knockdown and over-expression showed no significant difference in TNFα level. Thus, ERα possibly does not affect TNFα expression. However, over-expression of ERβ induced TNFα protein expression in MDA-MB-231 cells ( Figure 4C), and ERβ knockdown decreased TNFα level in MCF-7 cells ( Figure 4D). These results suggest that ERβ is likely to be the key regulator of TNFα expression.

Notably, high ERβ expression is significantly correlated with longer overall survival ( Figure 4E; *P*=0.0019) and disease-free survival ( Figure 4F; *P*=2.7e-09), indicating the biological significance of ERβ in breast cancer. The CCK8 assay demonstrated that *ERβ* is a tumor suppressor gene in breast cancer cells ( Figure 4G).

### Ginsenoside Rh2 induces TNFα expression via ERβ

**Figure 5:**
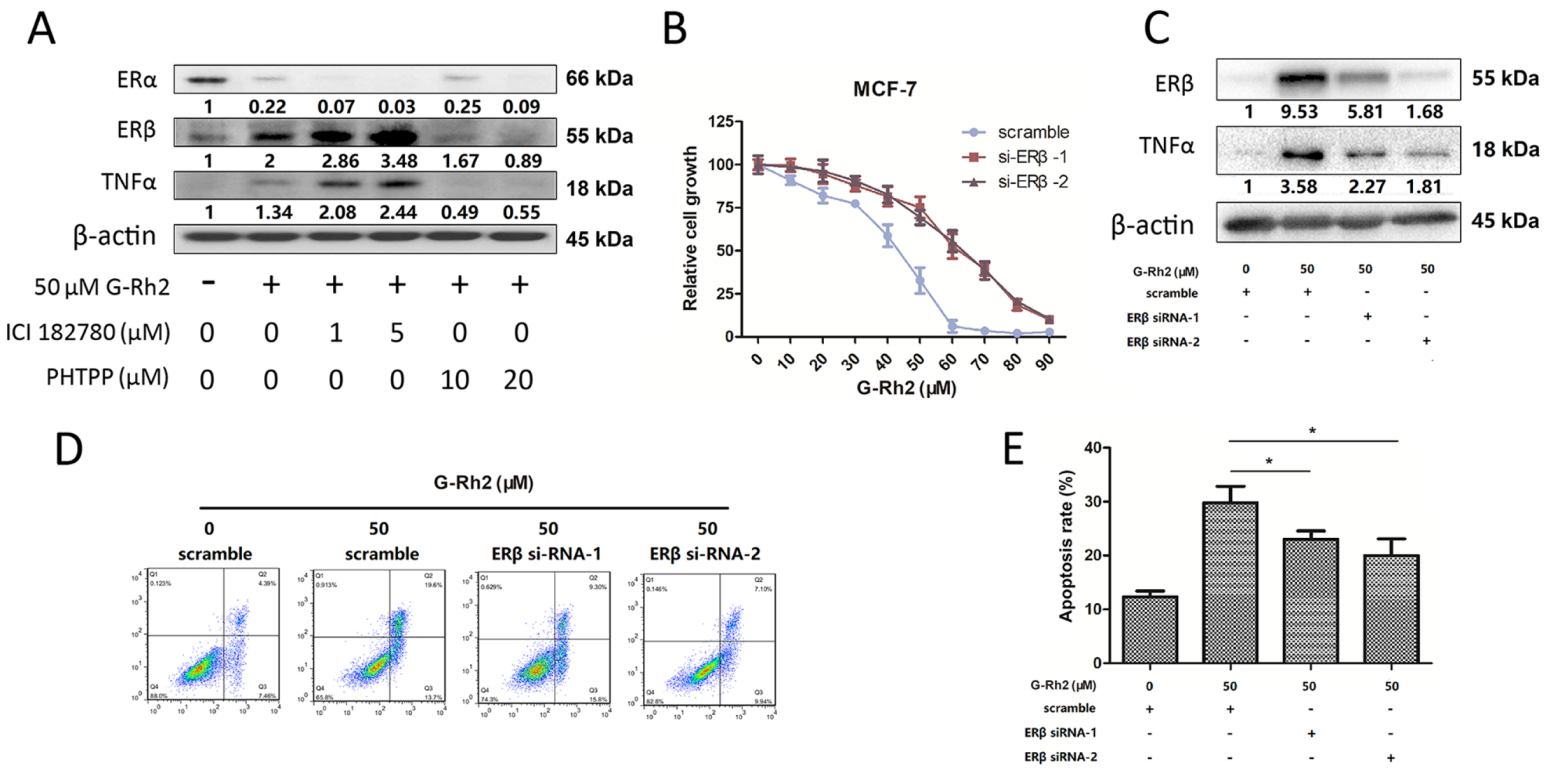
Figure5 Ginsenoside Rh2 induces TNFα expression via regulation of ERα and ERβ (A) MCF-7 cells were treated with 1 μM, 5 μM ICI182780 or 10 μM, 20 μM PHTPP in the presence or absence of 50 μM ginsenoside Rh2 for 48h, protein samples were collected for western blot analysis. (B) After transfection with ERβ siRNA, MCF-7 cells were treated with different concentrations of Rh2 for 48 h, and then subject to MTT assay. (C) The protein levels of ERβ and TNFα in MCF-7 cells were determined by western blot analysis. (D) Rh2-induced apoptosis of MCF-7 cells at 48 h after transfection with ERβ siRNA was determined by flow cytometry after PI/Annexin V staining. (E) Quantification of apoptosis. Data are presented as the mean±SD of three independent experiments. *P<0.05 versus control group.

ICI182780 is an ER antagonist, acting as a pure anti-estrogen by hindering receptor dimerization, increasing receptor turnover, and disrupting the nuclear localization of ERα [ 24, 25]. PHTPP is a selective ERβ inhibitor, which inhibits 17β-estradiol-stimulated ERβ activity but does not suppress 17β-estradiol-stimulated ERα activity [ 26, 27]. Using these ER inhibitors, we preliminarily investigated the effects of estrogen receptors on ginsenoside Rh2-induced over-expressions of TNFα. As shown in Figure 5A, ginsenoside Rh2, in synergy with ICI182780, down-regulated the protein level of ERα and increased the protein levels of ERβ and TNFα in a dose-independent manner, suggesting that down-regulation of ERα may enhance the accumulation of ERβ. Meanwhile, the ERβ inhibitor PHTPP lowered the protein level of ERβ, and inhibited TNF signaling in Rh2-treated MCF-7 cells. These results suggest that ERβ directly induces the expression of TNFα in Rh2-treated breast cancer cells. Moreover, after 48 h of incubation with various concentrations of ginsenoside Rh2, two ERβ siRNAs could rescue Rh2-induced cell inhibition with IC _50_ values of 61 μM, respectively, comparing to the scramble siRNA control with an IC _50_ value of 46 μM ( Figure 5B). Inhibition of ERβ effectively reversed the over-expression of TNFα induced by ginsenoside Rh2 ( Figure 5C), and rescued Rh2-induced apoptosis ( Figure 5D,E). These results suggest that ERβ plays a key role in Rh2-induced apoptosis.

### Ginsenoside Rh2 inhibits tumor growth of MCF-7 cells in vivo

**Figure 6:**
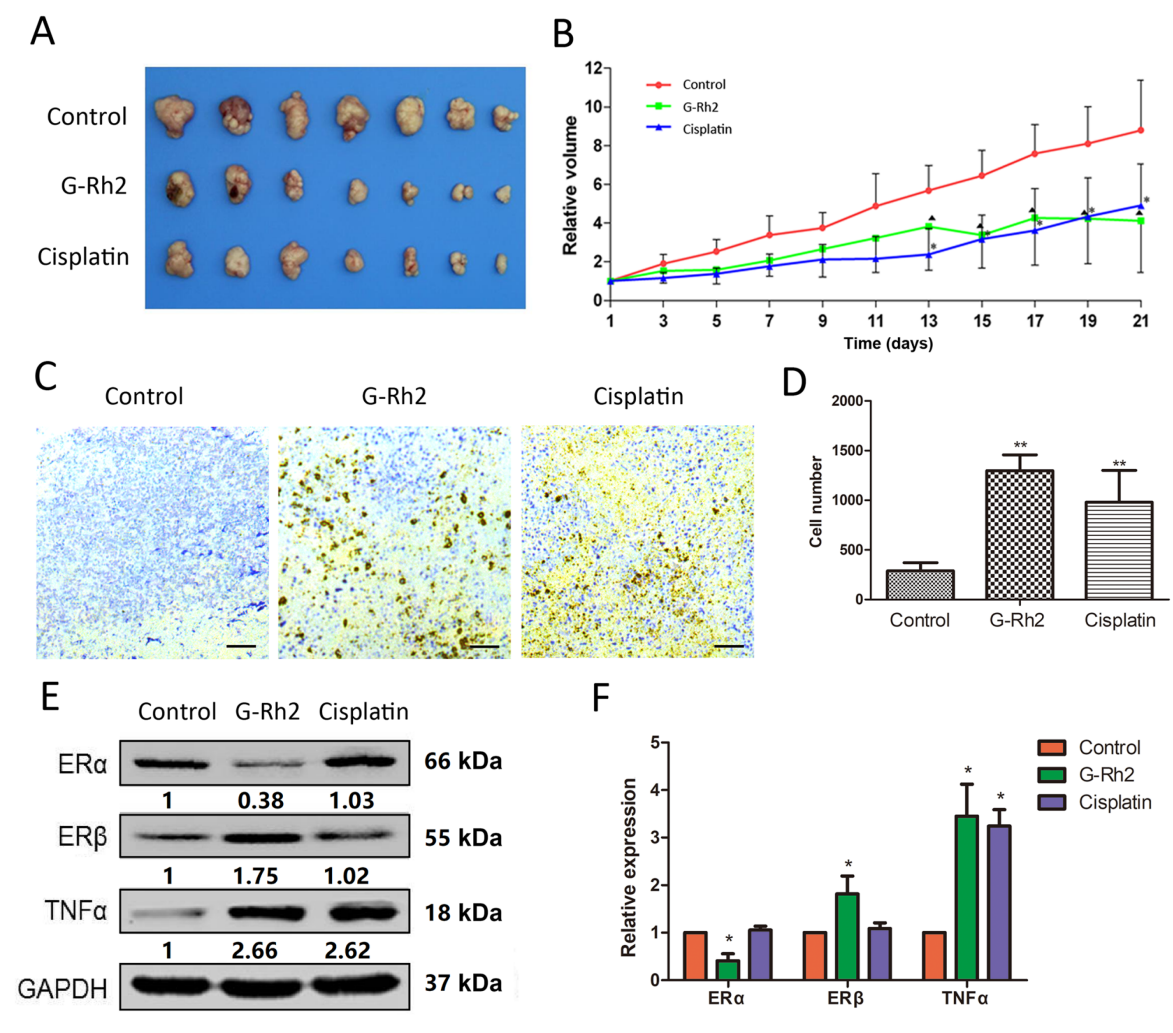
Figure6 Effects of ginsenoside Rh2 on tumor growth *in vivo* (A) Images of the dissected tumors are shown. (B) The volumes of the subcutaneous tumors were measured by caliper after implantation. Data are presented as the mean±SD. *P<0.05 and ΔP <0.05 versus control group, n=7 in each group. (C) TUNEL staining of tumor tissues from nude mouse xenograft model treated with ginsenoside Rh2 or cisplatin. Representative sections from each treatment group are shown. (D) TUNEL positive cells in tumor tissues were quantified. Scale bar=50 μm. **P<0.01 compared to controls, n=6 in each group. Data are presented as the mean±SD. (E) The protein levels of ERα, ERβ and TNFα in subcutaneous tumors were examined by western blot analysis. Expressions of proteins were quantified and normalized to the GAPDH loading control. Data are expressed as the mean±SD of three independent experiments. *P<0.01 versus control group.

Next, the effects of ginsenoside Rh2 on breast xenograft tumor growth were tested. As shown in Figure 6A,B, ginsenoside Rh2 and cisplatin (a positive control) significantly inhibited tumor growth after 13 days of treatment. The final xenograft tumors were also tested for apoptosis and expressions of ERα, ERβ and TNFα. As shown in Figure 6C,D, ginsenoside Rh2 and cisplatin significantly induced apoptosis. Ginsenoside Rh2 inhibited ERα expression, and induced the expressions of ERβ and TNFα ( Figure 6E,F). Comparing to ginsenoside Rh2, Cisplatin induced up-regulation of TNFα but had no effect on the expressions of ERα and ERβ.

## Discussion

Estrogen signaling occurs through both genomic and non-genomic pathways in which ERs regulate transcription of target genes directly or indirectly [14]. Upon induction by appropriate ligands, ERs form both homodimers and heterodimers which mediate target gene regulation through binding directly to EREs or tethering to EREs through transcription factors like AP-1 at their cognate response elements [28]. In addition, When ERα and ERβ are co-expressed in the cells, ERβ can antagonize ERα-dependent transcription [29]. Moreover, expression of ERβ increases proteolytic degradation of ERα [30]. Collectively, the ERβ-mediated inhibition of ERα activity involves a combination of altered recruitment of key transcription factors and increased ERα degradation. Specific ligands may induce conformational changes of estrogen receptors that preferentially recruit specific cofactors, thereby inducing differential responses [31]. Thus, 17β-estradiol can activate the ER signaling which is crucial for breast cancer progression, but ICI 182780, which is a derivative of 17β-estradiol with an added alkyl-sulfinyl moiety that degrades ER and affects its ligand-independent functions, is used for the treatment of hormone receptor positive metastatic breast cancer [32]. Bazedoxifene which works at nano-molar level is able to antagonize the effects of estrogen, while 2-phenylacetamide which is used for the treatment of perimenopause syndrome, increased the expressions of ERα and ERβ in MCF-7 cells at micromolar level [ 33, 34]. Various ligands regulate different functions of estrogen receptor for complex physiological processes.

Ginsenosides have some similarity in chemical structure with 17β-estradiol, and it was reported that various ginsenosides show different activities on estrogen receptors [ 35– 37]. Ginsenoside Rh2 is a protopanaxadiol (PPD)-type ginsenoside which has one glucose moiety at the C3 hydroxyl of PPD [38]. Comparing to the other protopanaxadiol (PPD)-type ginsenosides, ginsenoside Rh2 shows stronger anti-cancer activity [39]. It has been reported that ginsenoside Rh2 may inhibit growth, induce apoptosis and restrict tumor invasion and metastasis in mammalian tumor cells. Based on yeast two-hybrid assay, an illuminating experiment showed that ginsenoside Rh2 exhibits moderate estrogenic activity and is a weak phytoestrogen, compared with holothurin A, holotoxin A1, cucumarioside A2-2, frondoside A, and plant glycoside cauloside C [20]. In this study, we found that ginsenoside Rh2 induced the degradation of ERα which is analogous to ICI 182780 as a selective estrogen receptor degrader (SERD), and induced over-expression of TNFα via regulation of ERβ. Notably, ginsenoside Rh2 is a potential estrogen receptor ligand that can mediate unique biological effects. These findings suggest that more than 100 ginsenoside compounds are potential multi-functional phytoestrogen.

Triple-negative breast cancers (TNBCs) that do not express the genes for estrogen receptor (ER), progesterone receptor (PR) or Her2, lack known targetable biomarkers with an overall poor prognosis [40]. Moreover, there have been little advances in the treatment of TNBC compared with other subtypes [41]. About 20% of TNBC samples showed strong expression of nuclear ERβ which is of potential clinical interest [19]. In this study, we showed that ginsenoside Rh2 can initiate TNFα-induced apoptosis of breast cancer cells via ERβ. Ginsenoside Rh2 also effectively reduces the viability of TNBC MDA-MB-231 cells. Meanwhile, up-regulated expression of ERβ, a tumor suppressor, and ERβ-mediated accumulation of TNFα were also observed in TNBC MDA-MB-231 cells. These findings suggest that Rh2 may induce apoptosis via regulation of ER-TNFα pathway and may provide a novel potential strategy for cancer therapy.

Tumor necrosis factor (TNF) was identified as a cytotoxic product of immune cells, which is a cancer immunotherapeutic and causes lysis of tumor cells [ 12, 42]. Based on a previous study, the survival rate of Rh2-treated cells can be increased by blocking TNFα, proving that TNFα plays a key role in ginsenoside Rh2-induced apoptosis [10]. Upon binding to tumor necrosis factor receptor 1 (TNFR1) which is expressed in most tissues, TNFα is able to induce apoptosis through activating caspase-8 [43]. In addition, p38MAPK is activated by the recruitment of receptor-interacting protein (RIP) through tumor necrosis factor receptor type 1-associated DEATH domain protein (TRADD) in TNF signaling [44]. It was suggested that Rh2-induced apoptosis and G1/S phase arrest may be the results of the activation of TNF signaling. It is known that over-expression of ERβ enhances the accumulation of TNFα. Interestingly, there is no estrogen responsive elements (EREs) in the promotor sequence of TNFα, suggesting that nuclear receptor ERβ cannot directly bind to the promotor of TNFα, but may interact with the promoter by forming a complex with other transcription factors.

**Figure 7:**
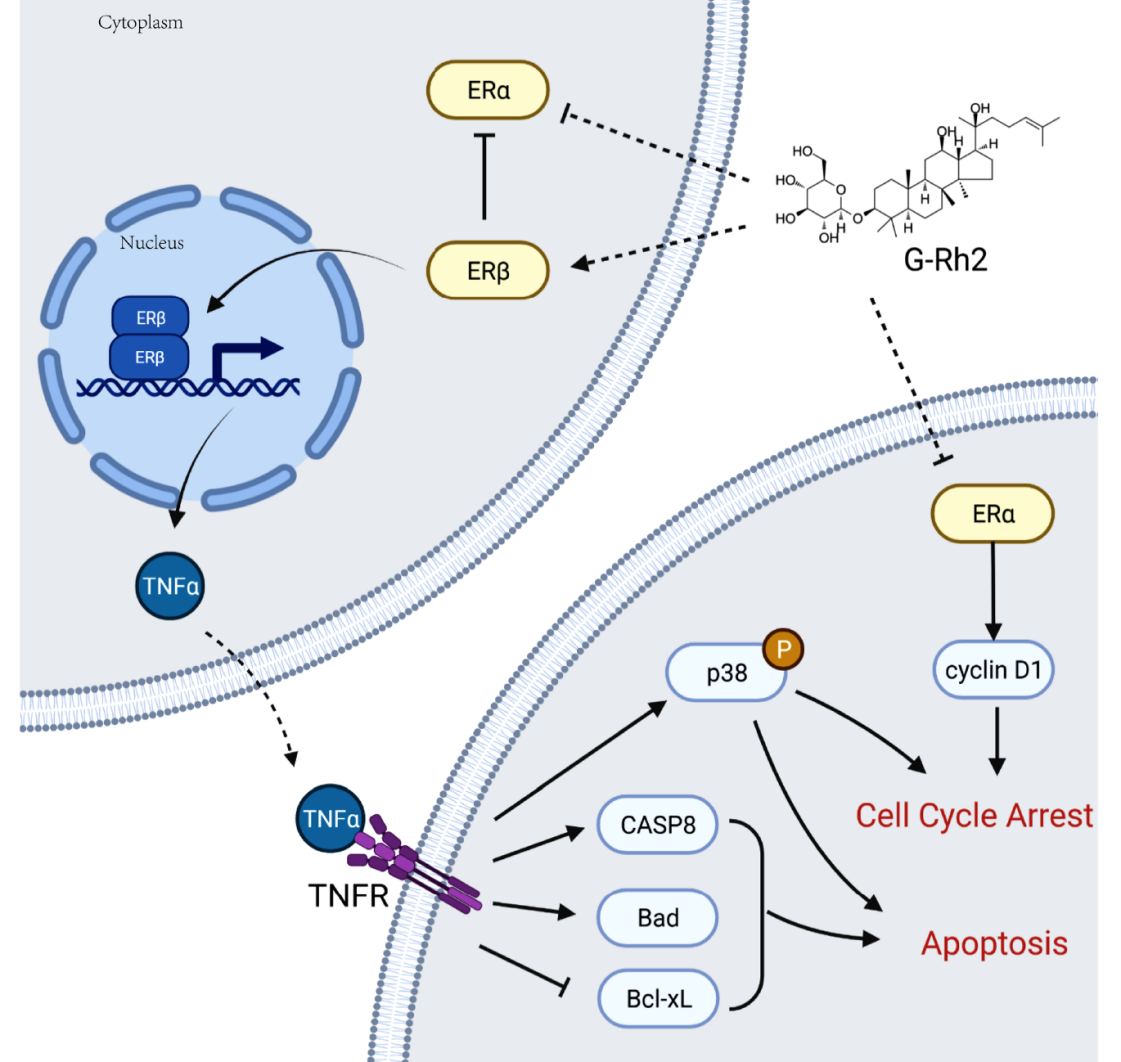
Figure7 Schematic diagram of the inhibition of breast cancer cell growth by Ginsenoside Rh2 Ginsenoside Rh2 induces over-expression of ERβ and down-regulation of ERα Over-expression ERβ enhances the transcription of TNFα. Secreted TNFα activates TNFR signaling pathway to inhibit breast cancer cell growth. Down-regulation of ERα contributed to G1/S phase arrest via inhibition of cyclin D1.

Ginsenoside Rh2 exhibits remarkable anticancer activity in various cancer cell lines, but the underlying molecular mechanisms of ginsenoside Rh2 are unclear [ 8, 39, 45– 47]. In general, ginsenoside Rh2 induces Bcl-2 family proteins-mediated apoptosis [48]. It has been reported that ginsenoside Rh2 upregulates long noncoding RNA to suppress breast cancer cell proliferation [49]. A recent study indicated that ginsenoside Rh2 is a major contributor to apoptosis through the mitochondrial pathway [50]. In this study, we found that ginsenoside Rh2 can regulate ERβ and ERα which are transcription factors involved in the regulation of many complex physiological processes. Figure 7 shows that ginsenoside Rh2 induces over-expression of ERβ and down-regulation of ERα. It was reported that ERβ can antagonize ERα [29]. Our previous data showed that over-expression of ERβ can increase the protein level of TNFα [10]. TNFα is a key protein in ginsenoside Rh2-induced cell growth inhibition. When over-expressed TNFα are secreted, TNFα receptors (TNFRs) activate distinct signaling pathways and induce the cellular behaviors. Apoptosis and cell cycle arrest will occur during this process when some downstream proteins of TNFRs, such as Caspase-8, Bad and p38, are activated and accumulated and the proteins like Bcl-XL are inhibited [13]. In addition, down-regulation of ERα also contributes to G1/S phase arrest via inhibition of cyclin D1 [ 23, 51].

Taken together, our data indicate that ginsenoside Rh2 induces breast cancer cell apoptosis and G1/S phase arrest. Moreover, ginsenoside Rh2 enhances the expression of TNFα via up-regulation of estrogen receptor β.
